# Properties of GPS noise at Japan islands before and after Tohoku mega-earthquake

**DOI:** 10.1186/2193-1801-3-364

**Published:** 2014-07-16

**Authors:** Alexey Lyubushin, Pavel Yakovlev

**Affiliations:** Institute of Physics of the Earth, Russian Academy of Sciences, Moscow, Russia; Russian State Geological Prospecting University, Moscow, Russia

**Keywords:** GPS, Time series analysis, Orthogonal wavelet transform, Spectral index

## Abstract

The field of 3-components GPS signals is analyzed for the network of 1203 stations at the Japanese islands from January 30 up to March 26, 2011. This time interval includes just over 40 days of observation before the Tohoku mega-earthquake on March 11, 2011 (M = 9.0) and nearly 16 days of observation following this event. The signals from each station are three-component time series with time step 30 minutes. We study the statistical properties of the random fluctuations of GPS signals before and after the seismic catastrophe after transition to increments. The values of wavelet-based spectral index for GPS noise components for each station were estimated separately for pieces of records before and after seismic event. The maps of the noise spectral index are constructed as the values for grid size of 50 × 50 nodes covering the region under study, based on information from 10 stations closest to each node. These maps clearly extract the region of future seismic catastrophe by relatively high noise spectral index. The using of principal components method distinguished this spatial anomaly more explicitly. These results support the hypothesis that statistical properties of random fluctuations of geophysical fields carry important information about earthquake preparation.

## Introduction

The noise properties of GPS signals is an object for investigation for a long time already. The shape of GPS power spectra and their spectral indexes were investigated in papers (Langbein & Johnson 1997; Zhang et al. 1997; Mao et al. 1999; Blewitt & Lavallee 2002; Williams et al. 2004; Wang et al. 2012). Correlations of GPS noise in temporal and space domains were investigated in (Beavan 2005; Teferle et al. 2008). The detail statistical structure of GPS time series was studied in (Li et al. 2000; Langbein 2008; Bos et al. 2008, 2010; Bock et al. 2011; Chen et al. 2013; Hackl et al. 2013; Goudarzi et al. 2013). In (Khelif et al. 2013) the GPS time series were investigated with the help of discrete wavelet transform for estimating positioning stability of stations and noise variance.

In this paper, the method for investigating properties of noise based on creating of maps of the noise characteristic of geophysical fields, which was developed in (Lyubushin 2012, 2013a, 2013b, 2014) for low-frequency seismic noise analysis, is used. It is applied to GPS signals on a network of stations, covering the entire territory of Japan. Analysis is performed for the random fluctuations of signals which are generated by transition to increments and is based on estimating of spectral index with the help of orthogonal wavelet expansions.

### Data

**Figure 1:**
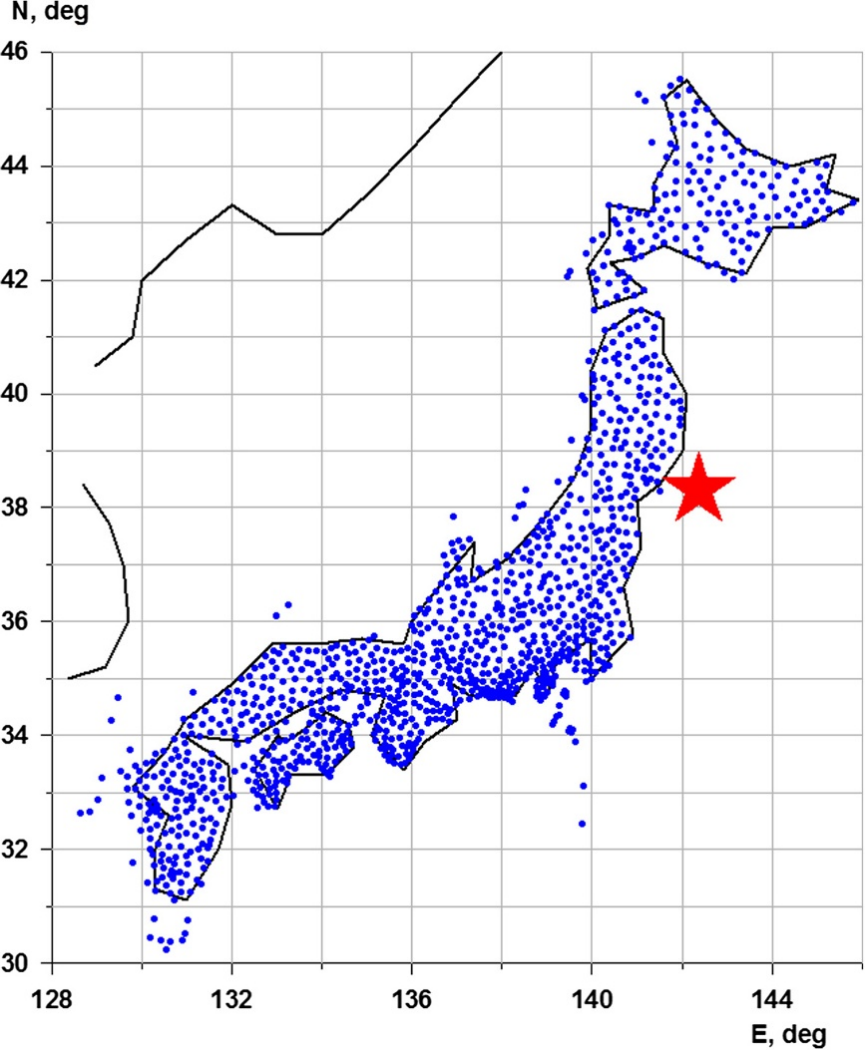
**Positions of GPS stations (blue points) and hypocenter of Tohoku earthquake on 11 March 2011 (red star).**

**Figure 2:**
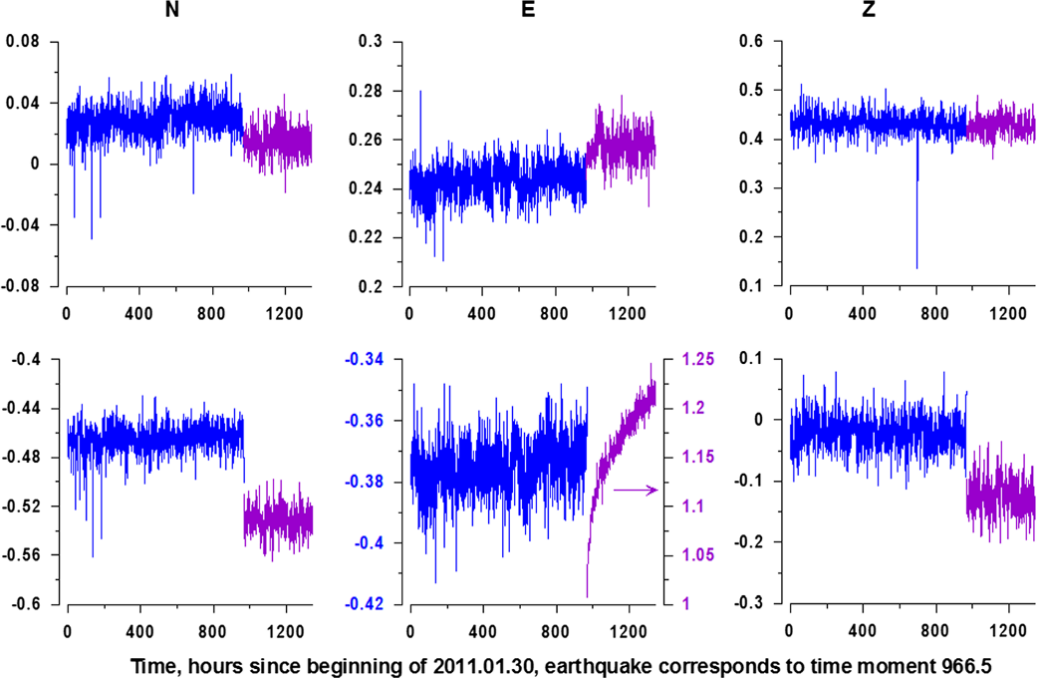
**Graphics of GPS time series before (blue lines, 1932 samples with 30-minutes time step) and after (purple lines, 756 samples) Tohoku earthquake.** Upper row of graphics corresponds to GPS station which is located far from hypocenter whereas lower row of graphics – to the GPS station which is near hypocenter.

The data present three-components GPS time series (N - offset to the north , E - offset to the east and Z - upward shift) with a sampling time step of 30 minutes. For the interval of observations from 30 January 2011 up to 26 March 2011 data can be freely downloaded from the address: http://quakesim.org/tools/timeseries.This time interval includes the mega-earthquake on March 11, 2011 (M = 9.0). Before the earthquake the length of the time series equals 1932 samples (a little over 40 days), after the seismic event the length equals 756 samples (about 16 days). Figure 1 shows the location of 1203 network stations GPS, and Figure 2 - examples of time series graphics for two GPS stations. Unfortunately the data are available for free downloading for time fragment from 30 January 2011 up to 26 March 2011 only - that is why our analysis is restricted by this time interval.

### Wavelet-based spectral index

Let  be the wavelet coefficients of the analyzed signal *x*(*t*), *t* = 1, …, *L*, is the discrete time, expanded in a system of orthogonal finite basis functions. The superscript *k* is the number of the detail level of the wavelet expansion, and the subscript *j* indicates the center of the time vicinity. The greatest possible value *m* of the detail level number depends on the volume of the sample analyzed. Here, we used a dictionary of 17 wavelets: 10 Daubechies ordinary orthogonal wavelets ranging in order from 2 to 20 (the use of higher orders entails numerical instability) and 7 so-called “symlets”; the latter are modifications of the Daubechies wavelets in which the form of basic functions is more symmetric than in ordinary wavelets (Mallat 1998). Symlets possess the same properties of compactness, orthogonality, completeness, and smoothness as wavelets do; however, for orders of 2 to 6, they coincide with the ordinary orthogonal Daubechies basis, while orders of 8 to 20 reveal distinctions in the form of a basis function. For these reasons, we used 17 variants of orthogonal compact basis functions.

In choosing the optimal wavelet basis, the criterion of the entropy minimum in the distribution of the squared values of the wavelet coefficients:
1

is commonly used (Mallat 1998). Here *m* is the number of detail levels which are taken into consideration, *M*_*k*_ is the number of wavelet coefficients at the detail level with number *k*. The value of *m* depends on the length *L* of the signal. For instance if *L* = 2^*n*^ then formally *m* = *n*, *M*_*k*_ = 2^(*n* - *k*)^. The condition *L* = 2^*n*^ is necessary for applying fast discrete wavelet transform (Mallat 1998). If the length *L* does not equal the power of 2, then the signal *x*(*t*) is appended by zero values up to the minimum integer number *N* which equals power of 2 and exceeds the length *L*: *N* = 2^*n*^ > *L*. At this case among the number 2^(*n* - *k*)^ of all wavelet coefficients at the detail level with number *k* only *L* ⋅ 2^- *k*^ corresponds to real signal variations whereas all other wavelet coefficients equal zero because of zero appending. Thus, in the formula (1) *M*_*k*_ = *L* ⋅ 2^- *k*^ and only “real” wavelet coefficients  are used for entropy computing.

Method (1) selects a basis for the signal *x*(*t*) such that the distribution of the signal wavelet coefficients differs most from a uniform distribution. In this case, maximum information concentrates in the minimum number of wavelet coefficients. After defining the optimal orthogonal wavelet basis from criterion (1) it is possible to calculate mean values of squared wavelet coefficients at each detail level:
2

Mean values (2) of squared orthogonal wavelet coefficients present the share of variance (energy of oscillations) corresponding to the frequency band of the detail level. It means that the value (2) could be regarded as wavelet-based power spectrum of the signal *x*(*t*). The frequency band of the detail level with number *k* is the following (Mallat 1998):
3

where Δ *s* is the length of the sampling time interval (in our case Δ *s* = 30 min). Let us consider the values of periods which correspond to central frequencies of the bands (3):
4

Thus, the value *S*_*k*_ = *S*(*T*_*k*_), *k* = 1, …, *m* is similar to usual Fourier power spectrum. The difference from classical Fourier spectrum estimates is that the values (2) are much more averaged – that is why the dependence *S*_*k*_ = *S*(*T*_*k*_) is much more smoothed. Let us consider the following model of the wavelet-based power spectrum (2):
5

where *ϵ*_*k*_ are white noise residual random values with zero mean. Parameter *b* in the formula (5) could be named a wavelet-based spectral index (or spectral exponent) and it is similar to usual spectral index which is widely used for investigating power spectra shapes. The value of *b* is estimated from least squares method: .

### Maps of spectral index for GPS noise at Japan islands

**Figure 3:**
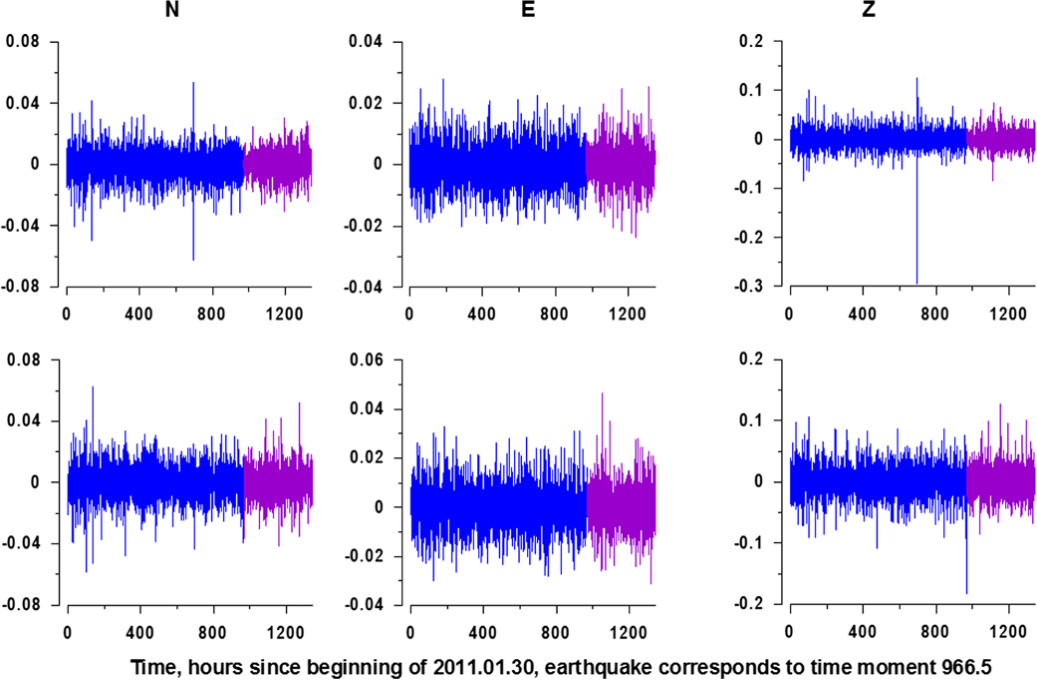
**Graphics of GPS time series increments (noise component) before (blue lines) and after (purple lines) Tohoku earthquake\ for the records presented at the Figure** 2 **.**

The values of spectral indexes *b* were calculated according to (5) for all 3-components records from GPS stations which are presented at the Figure 1 separately for their pieces before and after Tohoku earthquake (see Figure 2). Before estimating wavelet-based power spectra (2) the records were transformed to their increments. This operation strongly suppresses low-frequency components and amplifies high-frequency variations. Thus, coming to increments could be regarded as a noise extracting procedure. Figure 3 presents the increments of GPS records from Figure 2.

**Figure 4:**
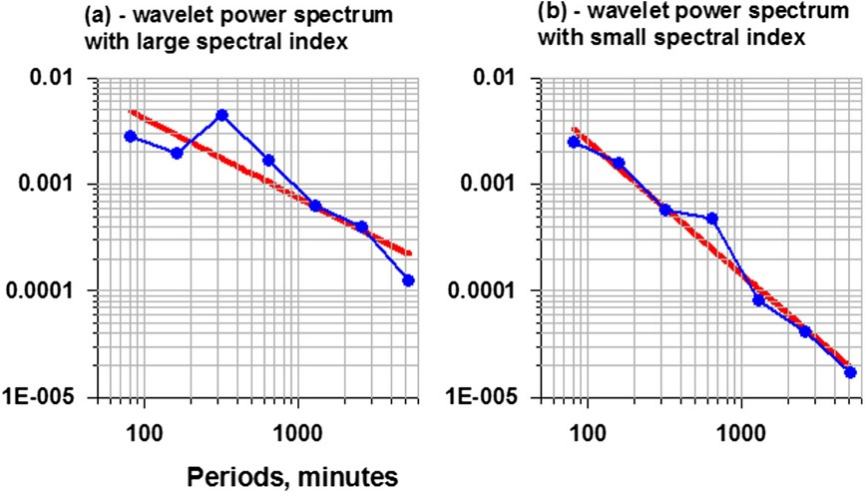
**Graphics of wavelet-based power spectra (blue lines) after coming to increments for 2 examples of East GPS time series before Tohoku earthquake.** Red lines present best fit lines in double logarithmic axes. Spectral index correspond to slope of red lines. Case **(a)** present large (taking into account the sign of the value) spectral index, whereas case **(b)** corresponds to small index.

Thus, the main purpose of the analysis was the properties of the GPS noise. Figure 4 presents examples of graphics of 2 wavelet-based power spectra with large and small values of spectral indexes *b*. The spectral indexes values are negative because of the operation of coming to increments which strongly suppresses low-frequency harmonics of the signal.

Having the values of *b* from all stations it is possible to create maps of spatial distribution of this statistic. For this purpose let us consider the regular grid of the size 50 × 50 nodes covering the rectangular domain with latitudes between 30°N and 46°N and longitudes between 128°E and 146°E (see Figure 1). For each node of this grid the values of *b* are corresponded which are calculated as median for the values of 10 nearest to the node GPS stations. This simple procedure provides the map. Taking into account that almost all stations of the F-net are placed at Japanese islands these map in the ocean regions have the less significance than at islands of course. It is evident the area for spatio-smoothing of spectral indexes is rather wide than data point distribution. This is a typical problem in geostatistics when it is necessary to extrapolate maps outside the region with stations of measurement. But we had to work with those data which we have at our disposal. The method of nearest neighbors which is used in this paper provides a rather natural extrapolation of the used values into domains which have no points of observations.

**Figure 5:**
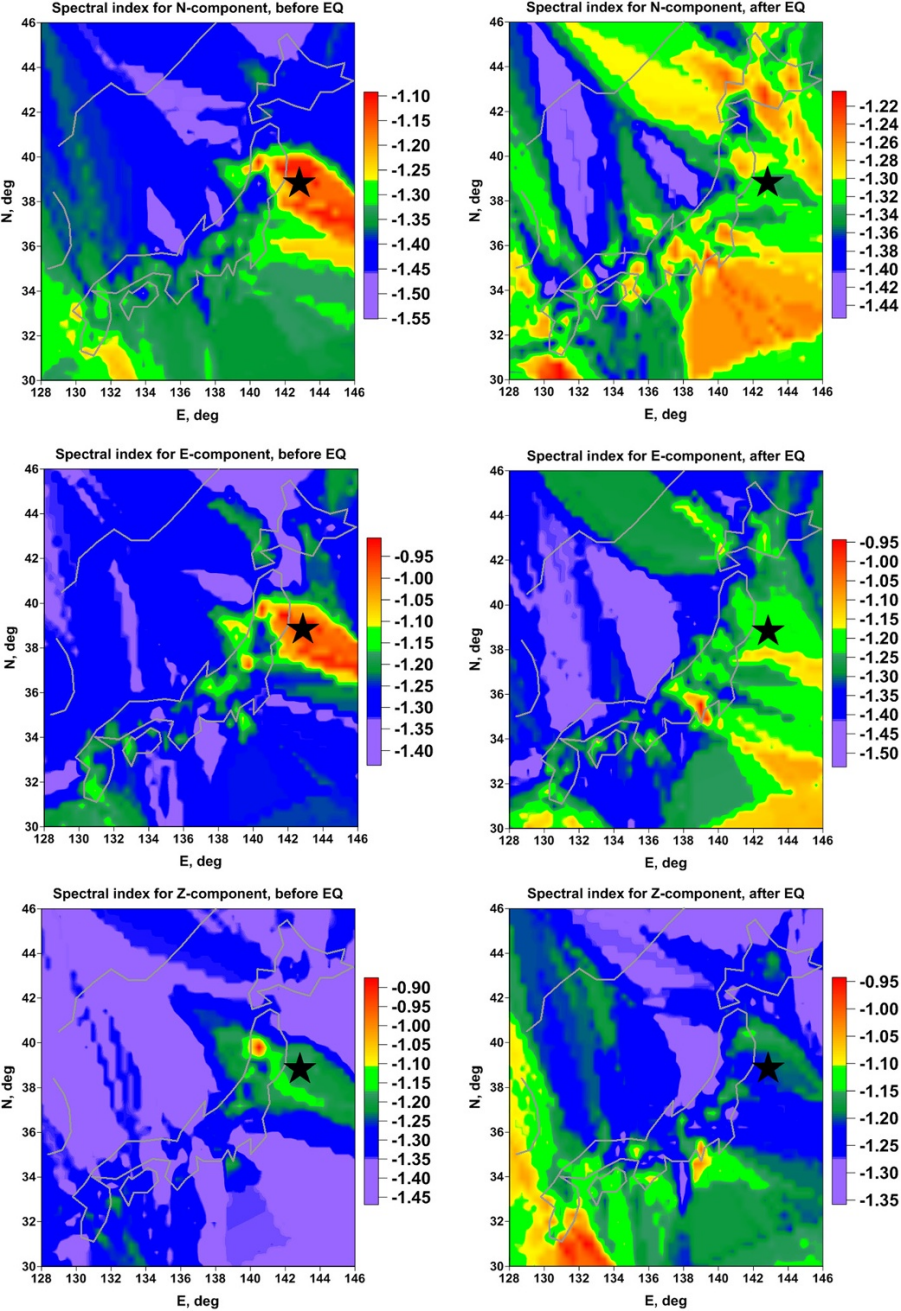
**Maps of wavelet-based spectral indexes for each GPS component before (left column of maps) and after (right column of maps) Tohoku earthquake.**

The Figure 5 presents maps of spectral index for all 3 components of GPS records for time intervals before and after Tohoku earthquake. It could be clearly noticed that the region of future seismic event is distinguished by relatively high values of spectral index for all components of GPS records.

### Maps of the first principal component

Let us apply the principal component method (Rao 1965) to the maps of spectral index which are presented at the Figure 5 in order to make more explicit common peculiarities of the spatial distributions for different components of GPS records.

Let us numerate by integer index *α* = 1, 2, 3 components E, N and Z correspondently. Let  be values of spectral index for GPS components in the node (*i*, *j*) of the regular grid, *i* = 1, …, *N*_*x*_; *j* = 1, … *N*_*y*_. In our case *N*_*x*_ = *N*_*y*_ = 50. Estimates of mean values and variances:
6

Let us consider correlation matrix of the size 3 × 3:
7

where . First principal components values in the nodes (*i*, *j*) are calculated by formula:
8

where *U*_*α*_ are components of eigenvector of the matrix *R* corresponding to its maximum eigenvalue.

**Figure 6:**
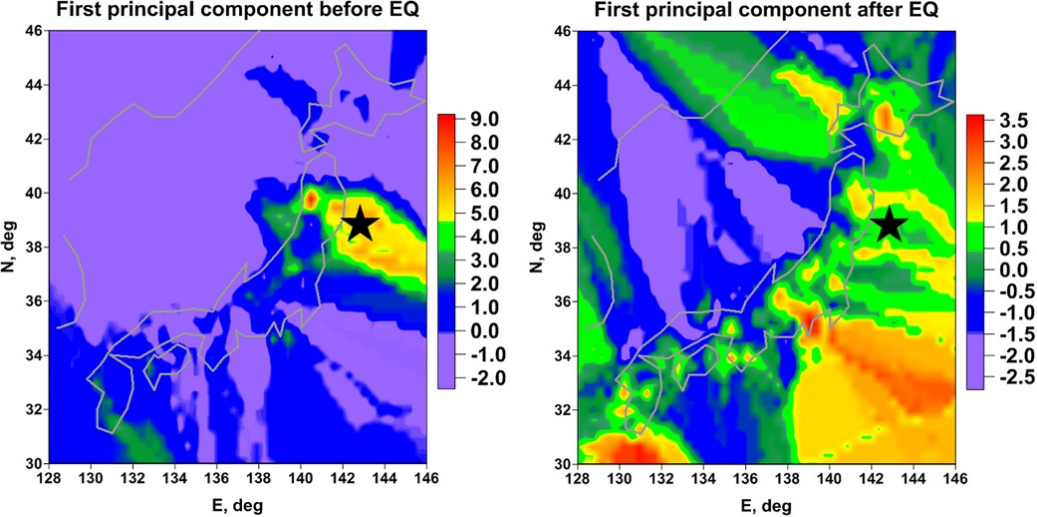
**Maps of the first principal component of 3 wavelet-based spectral indexes of all GPS components before (left map) and after (right map) Tohoku earthquake.**

The Figure 6 presents maps of 1^st^ principal component of spectral index for all components of GPS records for time intervals before and after Tohoku earthquake. We see that after applying principal components approach the spatial anomaly in the region of the future earthquake became much more explicit.

## Discussion

Investigation of the characteristics of random fluctuations of complex nonlinear systems is one of the most promising areas of research. This kind of research is at the intersection of different discipline, as this area has more common features than differences due to the specific characteristics of the objects under study. In this sense, the study of such a complex system as the planet Earth is no exception.

For example, low-frequency seismic noise has a complex statistical structure that encapsulates information about the preparation of geo-catastrophes, including major earthquakes, volcanic eruptions, activation aseismic movements, avalanches and landslides. Recent studies of seismic noise led to understanding that their statistical characteristics (mainly multi-fractal properties) contain the most valuable prognostic information. It was possible, in particular, to give (and publish a series of articles and abstracts on international conferences in 2008-2010) the forecast of mega-earthquake in Japan March 11, 2011, M = 9. The history of this prediction is described in details in (Lyubushin 2012, 2013a, 2013b, 2014).

In this paper we deal with other frequency range and with noise of other origin. It is known that GPS noise is generated by variations of conditions of atmosphere, points of observation and number of satellites, changing of snow cover and seasonal variations. We are interesting in peculiarities of spatial distribution of statistical properties of variations of points of observation, i.e. in the noise of “plates trembling”. From this point of view such large-scale variations which are connected with changes of satellites numbers, atmospheric and seasonal changes have an influence at all GPS stations simultaneously and they do not influence on extraction of spatial peculiarities of spectral index distribution. Besides that long-periodic variations are strongly suppressed by coming to increments.

## Conclusion

The main result of this paper which is presented at Figures 5 and 6 confirm the hypothesis that statistical properties of random fluctuations of GPS signals carry important information about earthquake preparation as well. This study gives a positive answer to the question "Could GPS be used to predict earthquakes?”.
